# Integrated Probiotic Benefits of *Bacillus velezensis* AAHM-BV2302 Drive Growth, Antioxidant Enhancement, and Immune Protection Against *Streptococcus agalactiae* in Tilapia (*Oreochromis* spp.)

**DOI:** 10.3390/antiox14111356

**Published:** 2025-11-13

**Authors:** Pakapon Meachasompop, Benchawan Kumwan, Putita Chokmangmeepisarn, Phornphan Phrompanya, Phunsin Kantha, Patcharapong Thangsunan, Prapansak Srisapoome, Pattanapong Thangsunan, Passakorn Kingwascharapong, Kentaro Imaizumi, Natthapong Paankhao, Kanokporn Saenphet, Supap Saenphet, Wararut Buncharoen, Anurak Uchuwittayakul

**Affiliations:** 1 Department of Aquaculture, Faculty of Fisheries, Kasetsart University, Bangkok 10900, Thailand; pakapon.meac@ku.th (P.M.); benchawan.kumw@ku.th (B.K.); phunsin.k@ku.th (P.K.); ffispssp@ku.ac.th (P.S.); 2 Center of Excellence in Aquatic Animal Health Management, Faculty of Fisheries, Kasetsart University, Bangkok 10900, Thailand; 3 Department of Microbiology, Faculty of Science, Kasetsart University, Bangkok 10900, Thailand; putita.cho@ku.th; 4 Department of Biological Science, Faculty of Science, Ubon Ratchathani University, Warin Chamrap District, Ubon Ratchathani 34190, Thailand; phornphan_ppy@hotmail.com; 5 Office of Research Administration, Chiang Mai University, Chiang Mai 50200, Thailand; tbscience@gmail.com; 6 Division of Biochemistry and Biochemical Innovation, Department of Chemistry, Faculty of Science, Chiang Mai University, Chiang Mai 50200, Thailand; pattanapong.t@cmu.ac.th; 7 Center of Excellence for Innovation in Chemistry, and Research Laboratory on Advanced Materials for Sensor and Biosensor Innovation, Material Science Research Center, Faculty of Science, Chiang Mai University, Chiang Mai 50200, Thailand; 8 Department of Fishery Products, Faculty of Fisheries, Kasetsart University, Bangkok 10900, Thailand; passakorn.ki@ku.th; 9 International Program in Bioscience and Technology, Faculty of Science, Kasetsart University, Bangkok 10900, Thailand; kentaro.im@ku.ac.th; 10 Kamphaeng Saen Fisheries Research Station, Faculty of Fisheries, Kasetsart University, Kamphaeng Saen Campus, Nakhon Pathom 73140, Thailand; ffisnpp@ku.ac.th; 11 Department of Biology, Faculty of Science, Chiang Mai University, Chiang Mai 50200, Thailand; kanokporn.saenphet@cmu.ac.th (K.S.); supap.saenphet@cmu.ac.th (S.S.)

**Keywords:** *Bacillus velezensis* AAHM-BV2302, probiotic, postbiotic, tilapia, antioxidant defense, cytokine expression, *Streptococcus agalactiae*, redox homeostasis, humoral immunity

## Abstract

Intensive aquaculture practices heighten oxidative stress and infectious disease risk, necessitating sustainable alternatives to antibiotics. This study evaluated the integrative probiotic and postbiotic potential of *Bacillus velezensis* AAHM-BV2302 in red tilapia (*Oreochromis* spp.), focusing on growth, antioxidant defense, immune modulation, and resistance to *Streptococcus agalactiae*. Whole-genome sequencing confirmed its classification as *B. velezensis* (4.16 Mb, GC 45.9%, ANI 99.4% with NRRL B-41580). Fish were fed diets supplemented with probiotic cells (Cell), cell-free supernatant (Cfs), or their combination (Cell + Cfs) for 30 days, followed by 30 days without probiotic supplementation. Growth performance significantly improved in Cell and Cell + Cfs groups at both Day 30 and Day 60 (*p* < 0.05). Antioxidant enzymes (SOD, CAT, GSH) increased significantly across tissues at Day 30, while malondialdehyde (MDA) declined (*p* < 0.05), indicating enhanced redox homeostasis. Humoral immunity was elevated, with higher lysozyme, bactericidal activity, and total IgM persisting post-supplementation (*p* < 0.05). Expression of *il1b*, *il6*, and *il8* was upregulated in immune-related and mucosal tissues, reflecting robust immune activation (*p* < 0.05). After *S. agalactiae* challenge, survival rates were 55% in Cfs, 60% in Cell, and 70% in Cell + Cfs, corresponding to relative percent survivals (RPS) of 43.8%, 50.0%, and 62.5%, respectively (*p* < 0.05). These results demonstrate that *B. velezensis* AAHM-BV2302 enhances growth, antioxidant capacity, and immune resilience through complementary probiotic–postbiotic mechanisms, supporting its application as a safe, multifunctional biotic for antibiotic-free tilapia aquaculture.

## 1. Introduction

Tilapia (*Oreochromis* spp.) ranks among the most extensively farmed freshwater fish species worldwide and in Thailand, valued for its rapid growth, adaptability to diverse environments, and strong consumer demand [1]. The hybrid red tilapia (*O. niloticus* × *O. mossambicus*) has further accelerated regional aquaculture development due to its superior growth performance, hardiness, and high market appeal [2]. However, the intensification of culture systems characterized by high stocking densities, nutrient enrichment, and deteriorating water quality, has led to a marked increase in the prevalence of infectious diseases [3,4]. Numerous bacterial and viral pathogens have been implicated in major outbreaks affecting tilapia, including *Streptococcus agalactiae*, *Edwardsiella tarda*, *Aeromonas hydrophila*, *A. veronii*, *Francisella orientalis*, *Plesiomonas shigelloides*, *Flavobacterium oreochromis*, and tilapia lake virus (TiLV). These pathogens often cause external and systemic infections manifested resulting in high mortalities and significant economic losses in both freshwater and brackish-water systems [5,6,7,8,9,10,11,12]. Among them, *S. agalactiae* induced streptococcosis remains one of the most devastating bacterial diseases in tilapia aquaculture worldwide, frequently responsible for severe mortality and recurrent outbreaks under intensive farming conditions.

To manage these infections, farmers traditionally rely on antibiotics and chemotherapeutic agents; however, such practices contribute to antimicrobial resistance (AMR), disrupt aquatic microbiota, and lead to drug residues in fish and the environment [13,14]. Moreover, oxidative stress induced by crowding, poor water quality, and pathogen infection impairs the activity of key antioxidant enzymes-superoxide dismutase (SOD), catalase (CAT), and glutathione (GSH), thereby exacerbating tissue damage and mortality [15]. These limitations emphasize the urgent need for sustainable, antibiotic-free interventions that can reinforce both immune and antioxidant defenses in cultured fish.

Probiotics have emerged as eco-friendly biotherapeutics that enhance fish health by modulating gut microbiota, strengthening mucosal barriers, and restoring redox homeostasis. In fish, intestinal microbes are central to digestion, nutrient assimilation, and immune regulation, and dysbiosis often precipitates inflammatory and oxidative disorders [16]. Dietary probiotics help restore microbial balance and augment enzymatic antioxidant capacity, thereby mitigating reactive oxygen species (ROS) accumulation and improving disease resistance [17]. Among bacterial probiotics, *Bacillus* species are particularly promising because of their spore-forming ability, which ensures survival during feed processing and gastrointestinal passage [18,19]. Moreover, *Bacillus* spp. produce extracellular enzymes and secondary metabolites—including lipopeptides (surfactin, fengycin, iturin) and polyketides (difficidin, bacillaene)—that exhibit antimicrobial, anti-inflammatory, and antioxidant activities, supporting growth and immune performance [20,21,22]. Previous studies have demonstrated that *Bacillus* supplementation enhances growth, antioxidant enzyme activities, and resistance to pathogens in freshwater species such as tilapia, catfish, and carp [23,24,25,26].

Recently, postbiotics—non-viable microbial components or metabolites that confer health benefits—have gained increasing attention as safe and stable complements to probiotics [27,28]. They can enhance growth, antioxidant status, and immune function without the viability limitations of live bacteria [29]. However, few studies have directly compared probiotic cells, their postbiotic cell-free supernatants (Cfs), and combined formulations in fish. Most previous research has focused solely on live probiotics or short-term feeding trials, without assessing the persistence of effects or responses across multiple tissues. Accordingly, the present study provides an integrative assessment of probiotic, postbiotic, and combined probiotic–postbiotic forms of *Bacillus velezensis* AAHM-BV2302, aiming to elucidate their complementary mechanisms and sustained benefits in tilapia aquaculture and representing the first integrated evaluation of probiotic (Cell), postbiotic (Cfs), and combined (Cell + Cfs) formulations with assessment of post-supplementation persistence across multiple tissues.

In this study, *Bacillus velezensis* has attracted increasing attention for aquaculture applications due to its strong antagonistic activity, ability to produce bioactive metabolites, and proven safety as a probiotic. The present study investigates the integrated effects of dietary *B. velezensis* AAHM-BV2302 isolated from healthy tilapia intestines on growth performance, antioxidant defense, humoral immune responses, pro-inflammatory cytokine expression, and resistance to *S. agalactiae*. By evaluating both probiotic (Cell) and postbiotic (Cell-free supernatant; Cfs) forms, this study elucidates how *B. velezensis* contributes to oxidative stress mitigation and immune enhancement, providing a mechanistic foundation for developing antioxidant-oriented, antibiotic-free probiotic strategies in tilapia aquaculture industry.

## 2. Methodology

### 2.1. Bacterial Cultivation and Preparation

*Bacillus velezensis* AAHM-BV2302 was propagated in tryptic soy broth (TSB) (Difco™, Franklin Lakes, NJ, USA) adjusted to pH 7.4 and incubated at 37 °C for 18–20 h until the mid-logarithmic phase (OD600 = 1.0), as verified by a UV–Vis spectrophotometer (Thermo Fisher Scientific, Waltham, MA, USA) against standard curves. Cell density was normalized to 1 × 10^9^ CFU/mL. Cultures were centrifuged at 5000× *g* for 8 min to obtain a cell-free supernatant (Cfs) and a bacterial pellet (Cell). The supernatant (Cfs) was used directly for feed top-dressing. The pellet was washed once with phosphate-buffered saline (PBS; pH 7.4) and resuspended to the original concentration (1 × 10^9^ CFU/mL) for probiotic use.

### 2.2. Whole-Genome Sequencing (WGS) and Taxonomic Identification

Genomic DNA libraries were prepared with the Illumina Nextera XT kit (San Diego, CA, USA) and sequenced on an Illumina HiSeq platform (paired-end). Raw reads were quality-checked and trimmed to remove adapters and bases with Q < 20 using Trimmomatic v0.39 [30]. High-quality reads were assembled with SPAdes v4.0.0 [31]. Draft genomes were annotated using Prokka v 1.14.5 for gene prediction and functional annotation [32]. Circular genome map was generated by Proksee web server (https://proksee.ca/ accessed on 10 May 2025) [33].

The *B*. *velezensis* AAHM-BV2302 genome was submitted to the Type Strain Genome Server (TYGS; https://tygs.dsmz.de accessed on 20 May 2025) to determine the closest type strains and digital DNA–DNA hybridization (dDDH) values [34]. Pairwise average nucleotide identity (ANI) and nucleotide similarity were calculated using JspeciesWS (version 5.0.2, https://jspecies.ribohost.com/jspeciesws accessed on 15 May 2025). A maximum-likelihood phylogeny based on WGS was inferred with IQ-TREE (1000 ultrafast bootstraps) [35]. The WGS-based phylogenetic tree was rendered in Interactive Tree of Life (iTOL) v.7 (https://itol.embl.de accessed on 10 May 2025). Additional phylogenetic analyses with *Bacillus* reference genomes retrieved from NCBI were conducted to confirm species-level resolution and evolutionary placement.

### 2.3. Effect of Dietary Supplementation with Probiotic B. velezensis AAHM-BV2302 on Tilapia Growth, Health, and Disease Resistance

#### 2.3.1. Ethical Statement

All experimental procedures involving aquatic animals were performed in compliance with the Ethical Principles and Guidelines for the Use of Animals established by the National Research Council of Thailand for research purposes. The study protocol was reviewed and approved by the Animal Ethics Committee of Kasetsart University, Thailand. (Approval ID: ACKU67-FIS-009. Approval date: 20 May 2024.)

#### 2.3.2. Fish Husbandry

A total of 400 healthy red tilapia (*Oreochromis* spp.) with an average body weight of 30.0 ± 5.0 g were procured from the Kamphaeng Saen Fisheries Research Station, Faculty of Fisheries, Kasetsart University, Kamphaeng Saen Campus, Nakhon Pathom, Thailand. Following collection, the fish were acclimated for 10 days at the Center of Excellence in Aquatic Animal Health Management, Department of Aquaculture, Faculty of Fisheries, Kasetsart University, Bangkok, Thailand. They were kept in 3000 L freshwater tanks under continuous aeration for 24 h. During the acclimation period, fish were fed a commercial herbivorous floating pellet diet (CP 9932, Thailand) containing crude protein (≥15.5%), crude lipid (≥3%), crude fiber (≤10%), and moisture (≤12%). Feeding was conducted three times daily at 09:00, 12:00, and 17:00. Prior to the experimental trial, the initial biomass and mean body weight of fish were recorded to ensure uniformity among treatments. Meanwhile, water quality parameters were closely monitored on a weekly basis to maintain optimal conditions for tilapia growth. The measured parameters remained within acceptable ranges throughout the experiment, with total ammonia nitrogen (TAN) 0.03–0.09 mg/L, alkalinity 95.6–109.2 mg/L as CaCO_3_, dissolved oxygen (DO) 3.2–5.0 mg/L, temperature 28.89–29.11 °C, pH 7.86–7.92 and, nitrite 0.07–0.13 mg/L, in accordance with standard methods [36].

#### 2.3.3. Bacterial Preparation, Diet Formulation, and Experimental Design

The experimental diets were freshly formulated each day by top-dressing a commercial floating feed (CP 9932, Thailand; crude protein ≥ 15.5%, crude lipid ≥ 3%, crude fiber ≤ 10%, moisture ≤ 12%). The feeding trial followed a completely randomized design (CRD) comprising five treatment groups, each with 4 replicates of 20 fish (totaling 80 fish per treatment). Upon completion of the acclimation period, fish were randomly allocated into 250 L tanks containing 200 L of well-aerated freshwater, maintained under identical environmental conditions as previously described.

1. Control group: fish fed a basal diet top-dressed with 100 mL of PBS per kg of feed.
2. Control-TSB group: fish fed a basal diet top-dressed with 100 mL of sterile TSB per kg of feed.
3. Probiotic (Cell) group: fish fed a diet supplemented with 100 mL of *Bacillus velezensis* AAHM-BV2302 suspension (1 × 10^9^ CFU/mL) per kg of feed, yielding an approximate concentration of 1 × 10^8^ CFU/kg of feed.
4. Cfs group: fish were fed a diet supplemented with 100 mL of cell-free supernatant per kg of feed, derived from bacterial cultures adjusted to an optical density of OD_600_ = 1.0 (equivalent to 1 × 10^9^ CFU/mL) after removal of the bacterial cells.
5. Cell + Cfs group: fish were fed a diet supplemented with 100 mL per kg of feed of a mixture containing *Bacillus velezensis* AAHM-BV2302 cells and their corresponding cell-free supernatant, prepared from cultures adjusted to an optical density of OD_600_ = 1.0 (equivalent to 1 × 10^9^ CFU/mL).

Throughout the feeding trial, probiotic-supplemented diets were administered for 30 days at a feeding rate of 4% of body weight per day, divided into three equal meals at 09:00, 12:00, and 17:00. Following this initial phase, all groups were fed the supplemented basal diet (CP 9932) for an additional 30 days to evaluate any residual probiotic effects. In total, the experimental period lasted 60 days.

#### 2.3.4. Sample Collection

The effects of probiotic supplementation on the health performance of red tilapia were assessed over a 60-day experimental period comprising two sampling intervals. The feeding trial involved probiotic administration for the initial 30 days, followed by additional 30 days of feeding with the basal diet (CP 9932) to assess post-supplementation effects. Sampling was conducted at the end of the probiotic-feeding phase (Day 30) and again at the conclusion of the trial (Day 60). At each sampling point, eight fish were randomly chosen from each treatment group (two fish per replicate, totaling eight fish per group). For serum collection, non-anticoagulated whole blood was drawn from the caudal vein using sterile syringes. The samples were left to clot at room temperature for 1 h and subsequently centrifuged at 5000× *g* for 15 min. The separated serum was carefully collected and stored at −80 °C for subsequent biochemical analysis. Fish tissues were also sampled on Days 30 and 60 and immediately stored at −80 °C for determination of antioxidant enzyme activities and malondialdehyde (MDA) content. For gene expression analysis, intestinal samples were obtained for immune-related genes, while brain, muscle, and liver tissues were collected for growth-related gene expression. All tissue samples were aseptically handled and promptly immersed in 1.0 mL of TRIzol™ reagent (RNA-spin™ Total RNA Extraction Kit, iNtRON Biotechnology, Seongnam-si, Republic of Korea) following the manufacturer’s instructions.

#### 2.3.5. Growth Performance

Throughout the experimental period, fish were individually weighed one day before the trial commenced and subsequently on Days 30 and 60. To assess growth performance, red tilapia were randomly sampled from each replicate within all treatment groups (10 fish per replicate; 40 fish per group). The evaluated growth parameters included survival rate, weight gain (WG), average daily gain (ADG), total feed intake, and feed conversion ratio (FCR). All growth performance indices were determined according to the procedures described by [37].

1. The following formulas were applied.
2. Total weight gain (TWG, g/30 or 60 days) = *Wt* − *Wi*
3. Average daily gain (ADG, g/day) = (*Wt* − *Wi*)/t

where *Wt* is the final weight, *Wi* is the initial weight, and *t* is the duration of the trial (30 or 60 days).

4. Feed Conversion Ratio (FCR) was calculated as the ratio of the total feed share intake to the individual weight gain (WG): FCR = Total feed share intake/Weight gain (WG)

FCR for each fish was estimated by proportionally allocating the total feed intake of the tank based on each fish’s initial body weight.

#### 2.3.6. Determination of Oxidative Stress and Antioxidant Status in Target Organs

The tissues selected for antioxidant analysis included the spleen, head kidney, liver, serum, muscle, skin, intestine and gills. For sample preparation, approximately 50 mg of each tissue was accurately weighed and homogenized in 1 mL of phosphate-buffered saline (PBS; 0.1 M, pH 7.4). The homogenates were then centrifuged at 1100× *g* for 10 min at 4 °C, and the resulting supernatants were carefully transferred into microcentrifuge tubes. All supernatant samples were stored at −20 °C until further biochemical analysis.

(1) Measurement of malondialdehyde (MDA) levels

Malondialdehyde (MDA) concentration was assessed using the thiobarbituric acid reactive substances (TBARS) assay, as previously described [38,39] with minor modifications. In brief, 100 µL of the tissue supernatant was mixed with 450 µL of 0.85% (*w*/*v*) normal saline, 1000 µL of 10% (*w*/*v*) trichloroacetic acid (TCA), and 200 µL of 0.67% (*w*/*v*) thiobarbituric acid (TBA). The reaction mixture was then heated in a boiling water bath for 30 min and subsequently cooled to room temperature. After cooling, 2 mL of distilled water was added, followed by centrifugation at 1100× *g* for 10 min. The absorbance of the resulting supernatant was recorded at 532 nm using a UV–Vis spectrophotometer. MDA concentration was determined against a tetramethoxypropane (TMP) calibration curve and expressed as nanomoles per milligram of protein.

(2) Measurement of catalase (CAT) enzyme activity

Catalase (CAT) activity was measured according to the method of Maehly [40] with minor modifications. In brief, 100 µL of the tissue supernatant was combined with 2.5 mL of phosphate buffer (50 mM, pH 5.0) and 0.4 mL of hydrogen peroxide (5.9 mM). The decrease in absorbance at 240 nm was recorded using a spectrophotometer, with readings taken at 30 s intervals for 2 min. CAT activity was calculated relative to a catalase standard curve and expressed as international units per minute per milligram of protein (IU/min/mg protein).

(3) Determination of glutathione (GSH) content

Glutathione (GSH) concentration was determined according to the method of Jollow et al. [41] with minor modifications. Briefly, 1 mL of 4% (*w*/*v*) sulfosalicylic acid was added to 1 mL of the tissue supernatant, and the mixture was incubated at 4 °C for 1 h to precipitate proteins. Following incubation, the samples were centrifuged at 1100× *g* for 20 min at 4 °C, and the clear supernatant was collected. A 100 µL aliquot of this supernatant was subsequently mixed with 2.9 mL of reaction mixture containing 100 mM phosphate buffer (pH 7.4) and 100 mM 5,5′-dithiobis-(2-nitrobenzoic acid) (DTNB). The formation of a yellow-colored chromophore was monitored at 412 nm using a spectrophotometer. GSH content was quantified using a standard calibration curve and expressed as micromoles per milligram of protein (µmol/mg protein).

(4) Measurement of superoxide dismutase (SOD) enzyme activity

Superoxide dismutase (SOD) activity was measured based on the method of Paankhao et al. and Takada et al. [38,42] with minor modifications. In brief, 100 µL of the tissue supernatant was combined with 1 mL of reaction mixture containing 0.1 mM xanthine, 0.025 mM nitro blue tetrazolium (NBT), 0.1 mM disodium ethylenediaminetetraacetate dihydrate (EDTA·2H_2_O), 60 mM sodium carbonate buffer (pH 10.2), and xanthine oxidase. The rate of NBT reduction was monitored at 560 nm using a spectrophotometer, with absorbance readings taken every 30 s for 2 min. SOD activity was quantified using a standard calibration curve and expressed as milli-international units per minute per milligram of protein (mIU/min/mg protein).

#### 2.3.7. Humoral Innate Immune Responses Assays

(5) Lysozyme activity

Lysozyme activity was determined according to the method of Parry et al. [43] with slight modifications. Briefly, *Micrococcus lysodeikticus* (Sigma-Aldrich, Darmstadt, Germany) was suspended in phosphate-buffered saline (PBS, pH 6.2) to obtain a final concentration of 0.2 mg/mL, and the suspension was incubated for 1 h prior to use. Subsequently, 10 µL of fish serum was pipetted into each well of a flat-bottomed microtiter plate, followed by the addition of 250 µL of the *M. lysodeikticus* suspension. The absorbance at 540 nm was measured using a microplate spectrophotometer (iMark™ Microplate Absorbance Reader, BIO-RAD, Hercules, CA, USA) at 0 and 5 min during incubation at room temperature.

U/mL = (ΔA_450_/min × V*total*) ÷ (0.001 × V*sample*) × DF

where

ΔA_450_/min = change in absorbance per minute

V*total* = total reaction volume (mL)

V*sample* = volume of serum sample (mL)

DF = dilution factor of the sample (DF = 1 if no dilution was applied)

(6) Bactericidal activity

The bactericidal activity assay was performed according to the method described by [37] with minor modifications. A virulent strain of *Streptococcus agalactiae* was cultured in tryptic soy broth (TSB) under the conditions detailed in Section 2.1, and subsequently used to evaluate the bactericidal activity of serum samples. The bacterial suspension was standardized to 1 × 10^5^ CFU/mL in sterile phosphate-buffered saline (PBS, pH 7.4) based on the absorbance measurement at 600 nm. For the assay, 40 µL of each sample was mixed with 10 µL of the *S. agalactiae* suspension (1 × 10^3^ CFU/reaction) and incubated at room temperature for 2 h. After incubation, the mixtures were spread onto tryptic soy agar (TSA, Germany) and incubated for 24 h. The colony-forming units (CFU) were then enumerated, and bactericidal activity (BA) was calculated using the following equation:

BA (%) = [(T_0_ − T_24_)/T_0_] × 100

where *T*_0_ is the initial bacterial count and *T*_24_ is the number of viable bacteria remaining after 24 h of incubation.

(7) Total serum IgM

Serum immunoglobulin M (IgM) levels were determined using a direct ELISA technique, as described in previous studies [44,45], with slight modifications. Briefly, 96-well microplates were coated with bicarbonate/carbonate buffer (pH 9.6) and incubated for 2 h at room temperature (RT). After removal of the coating buffer, 100 µL of fish serum (diluted 1:100) was added to each well and incubated overnight at 4 °C. The plates were then washed three times with PBST (PBS containing 0.05% Tween-20, pH 7.4) and subsequently blocked with 100 µL of VisualProtein-BlockPRO™ Blocking Buffer for 2 h at RT. After another washing step, a rabbit anti-tilapia IgM monoclonal antibody (GeneScript, Piscataway, NJ, USA) was added and incubated for 2 h at RT. Following six additional washes, 100 µL of TMB One Component HRP Microwell Substrate was introduced into each well and incubated for 45 min at RT in the dark. The enzymatic reaction was terminated by adding 100 µL of TMB Stop Solution, and the absorbance was recorded at 450 nm using an iMark™ Microplate Absorbance Reader (Bio-Rad Laboratories Ltd., Hercules, CA, USA). Negative control wells (without fish serum) were included to determine the cutoff absorbance for data interpretation.

#### 2.3.8. Total RNA Isolation, First-Strand cDNA Synthesis and Gene Expression Analysis by Quantitative Real-Time RT-PCR (qRT-PCR)

Total RNA was extracted from head kidney, spleen, PBLs, liver, muscle, intestine, gills and skin using the RNA-spin™ Total RNA Extraction Kit (iNtRON Biotechnology, Seongnam-si, Republic of Korea), following the manufacturer’s instructions. The purity and concentration of RNA were determined using a NanoDrop™ spectrophotometer (Thermo Fisher Scientific, Waltham, MA, USA). A total of 1.0 µg of RNA from each sample was used as a template for first-strand complementary DNA (cDNA) synthesis using the Maxime™ RT PreMix kit (iNtRON Biotechnology, Seongnam-si, Republic of Korea). The synthesized cDNA was stored at −20 °C until further use.

The quantitative real-time PCR (qRT-PCR) was performed using QuantiNova SYBR Green PCR chemistry (Qiagen, Germany) on an Azure Cielo™ real-time PCR system (Azure Biosystems, Dublin, CA, USA). The amplification program included an initial denaturation at 95 °C for 5 min, followed by 40 cycles of 95 °C for 30 s, 60 °C for 30 s, and 72 °C for 90 s, with a final extension step at 72 °C for 10 min. The housekeeping genes (*actb1* and *rna18s*) were employed as internal controls to normalize mRNA and cDNA levels, ensuring the reliability and consistency of quantification. Target genes were amplified to evaluate the expression of non-specific immune-related and growth-related genes. All primers were validated for amplification efficiency prior to qRT-PCR analysis.

All primers were standardized to ensure amplification efficiency prior to the qRT-PCR analysis. The relative expression levels of target genes were calculated using the 2^−ΔΔCT^ method, following the procedure described by Livak and Schmittgen [46]. The primer sequences for all analyzed genes are listed in Table 1.

#### 2.3.9. Disease Resistance and Survival Rate (SR) Following *Streptococcus agalactiae* Challenge

At the end of the 60-day feeding trial, 20 fish from each treatment group (five fish per replicate) were randomly selected and transferred to 250 L fiberglass tanks equipped with continuous aeration.

The fish were experimentally challenged with *Streptococcus agalactiae* via intraperitoneal (*i.p.*) injection. The pathogen was cultured in tryptic soy broth (TSB; Difco™, Franklin Lakes, NJ, USA), and the bacterial density was adjusted using a spectrophotometer, as described in Section 2.1.

Each fish received an injection of 100 μL of *S. agalactiae* suspension containing 1 × 10^6^ CFU/mL, equivalent to 1 × 10^5^ CFU/fish. The challenge dose was selected based on preliminary LD_50_ trials conducted in red tilapia over a 14-day period. Bacterial concentrations were verified by plate counting before and after injection to ensure dose consistency. Fish mortality was monitored daily for 14 days, and survival rate (SR) was analyzed using the Kaplan–Meier method [49]. The relative percent survival (RPS) was calculated according to the following formula:

RPS (%) = [1 − (mortality in the treatment group ÷ mortality in the control group)] × 100

#### 2.3.10. Statistical Data Analysis

The statistical analysis was conducted using GraphPad Prism version 10.4.0 for macOS (GraphPad Software, Boston, MA, USA), applying a one-way analysis of variance (ANOVA) followed by Tukey’s multiple comparison test after passing the Shapiro–Wilk test to assess the normality of data distribution. Survival data were analyzed using the Kaplan–Meier method, and survival curves were generated in GraphPad Prism to illustrate survival patterns over time. All data are presented as mean ± standard deviation (SD). Different letter superscripts and asterisks (*) indicate statistically significant differences among groups, with a significance level of *p* < 0.05.

## 3. Results

### 3.1. Genomic Identification and Characterization of Bacillus velezensis AAHM-BV2302

**Figure 1:**
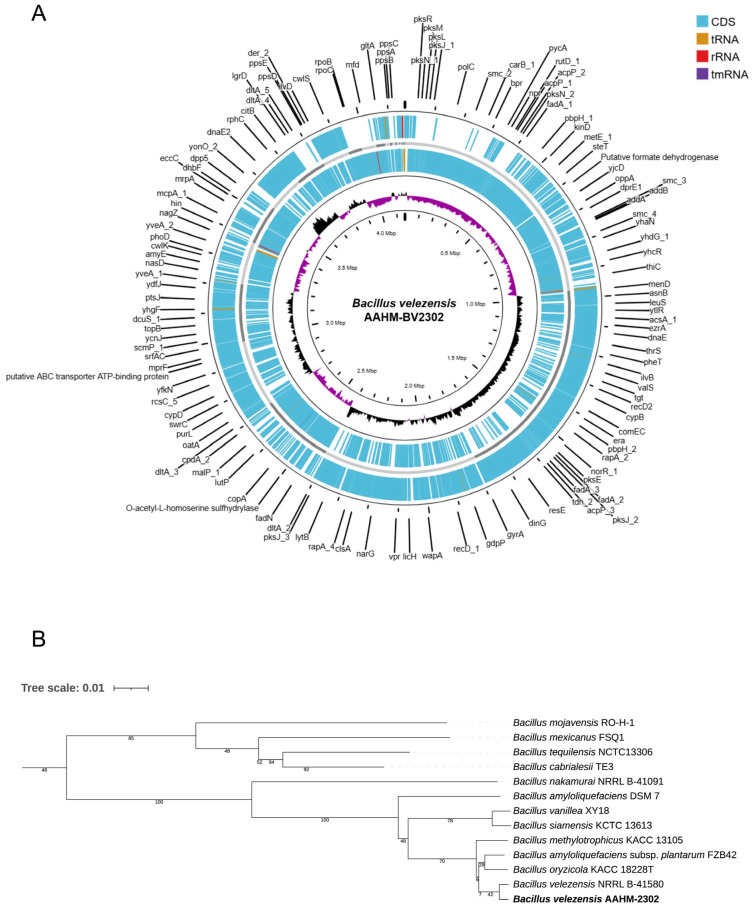
Genomic features and phylogenetic relationship of *Bacillus velezensis* AAHM-BV2302. (**A**) Circular representation of the draft genome showing coding sequences (CDSs), tRNAs, rRNAs, and tmRNA. The inner rings display GC content and GC skew. (**B**) Phylogenetic tree based on whole-genome sequences of *B. velezensis* AAHM-BV2302 and closely related *Bacillus* species, constructed using the neighbor-joining method. The assembled genome sequence has been deposited in NCBI GenBank under the accession number JBQXGX000000000 and BioProject accession number PRJNA1320929.

The complete genome of *Bacillus velezensis* strain AAHM-BV2302 was sequenced and successfully assembled into a single circular chromosome, spanning 4,156,630 base pairs with a GC content of 45.9% (Figure 1A). Annotation revealed 4177 genes, including 4103 coding sequences (CDSs), 69 tRNAs, four rRNAs, and one tmRNA (Table 2).

Phylogenomic analysis based on whole-genome sequences confirmed that strain AAHM-BV2302 clustered within the *Bacillus velezensis* clade, together with closely related reference strains (Figure 1B). The strain exhibited the highest genomic similarity with *B. velezensis* NRRL B-41580 (ANI = 99.43%, dDDH = 97.3%, nucleotide similarity = 93.42%) and *B. amyloliquefaciens* subsp. *plantarum* FZB42 (ANI = 98.04%, dDDH = 90.6%, nucleotide similarity = 87.09%). These values exceeded the accepted species delineation thresholds, thereby confirming the taxonomic assignment of AAHM-BV2302 as *B. velezensis*. Collectively, these results provide strong genomic evidence supporting the classification of AAHM-BV2302 as a distinct *B. velezensis* strain (Table 3). The assembled genome sequence has been deposited in NCBI GenBank under the accession number JBQXGX000000000, within the BioProject accession number PRJNA1320929.

### 3.2. Growth Parameters and Immune-Growth-Related Gene Expression

#### 3.2.1. Growth Performance

**Figure 2:**
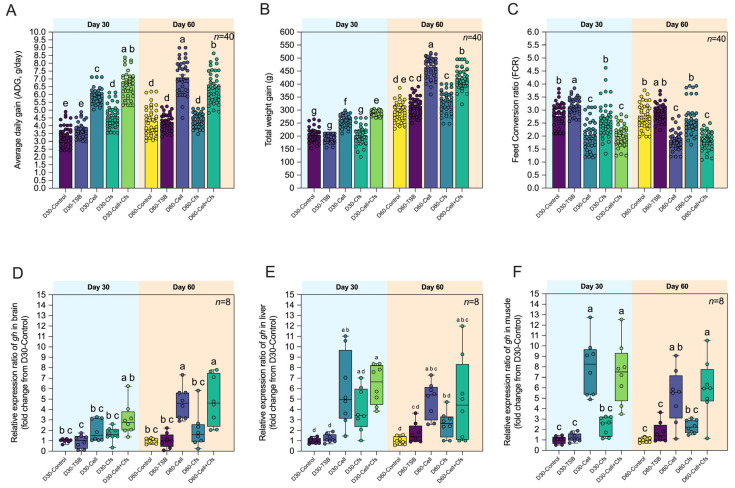
Growth performance and growth-related gene expression of tilapia following dietary supplementation with *B*. *velezensis* AAHM-BV2302. Parameters include average daily gain (ADG) (**A**), total weight gain (TWG) (**B**), feed conversion ratio (FCR) (**C**) (*n* = 40), and relative expression (fold change) of *gh* in brain (**D**), liver (**E**), and muscle (**F**) after 30 days of probiotic administration (Day 30) and 30 days post-supplementation (Day 60). Data are represented as mean ± SD (*n* = 8). Different letters above bars indicate statistically significant differences among groups (*p* < 0.05).

The effects of dietary probiotic supplementation on growth performance in tilapia are presented in Figure 2A–C. After 30 days of probiotic administration (D30), the D30-Cell + Cfs group exhibited the highest average daily gain (ADG; 6.61 ± 0.85 g day^−1^), followed by D30-Cell (5.91 ± 0.49 g day^−1^) and D30-Cfs (4.62 ± 0.72 g day^−1^), all of which were significantly higher than D30-Control (3.38 ± 0.67 g day^−1^) and D30-TSB (3.71 ± 0.48 g day^−1^) (*p* < 0.05). After 30 days post-supplementation (D60), ADG in D60-Cell and D60-Cell + Cfs remained significantly higher than in both controls (7.10 ± 1.03 and 6.48 ± 0.99 g day^−1^, respectively; *p* < 0.05), while D60-Cfs (4.37 ± 0.43 g day^−1^) remained comparable to controls (*p* > 0.05). For total weight gain (TWG), the D30-Cell + Cfs (288.83 ± 9.41 g) was significantly greater than D30-Control (204.40 ± 26.06 g) and D30-TSB (190.94 ± 14.19 g), followed by D30-Cell (260.21 ± 21.58 g) (*p* < 0.05), while the D30-Cfs (203.49 ± 33.91 g) showed no significant difference from controls (*p* > 0.05). At Day 60, probiotic-treated groups maintained significantly higher TWG values compared with D60-Control (293.27 ± 36.08 g), with D60-Cell, D60-Cfs, and D60-Cell + Cfs showing 465.57 ± 39.65, 331.87 ± 41.48, and 422.80 ± 41.05 g, respectively (*p* < 0.05), while D60-TSB (314.96 ± 35.49 g) was significantly lower than D60-Cell and D60-Cell + Cfs (*p* < 0.05), but not different from D60-Cfs (*p* > 0.05). Regarding feed conversion ratio (FCR), the D30-TSB group exhibited the highest value (3.14 ± 0.30) compared with D30-Control, D30-Cell, D30-Cfs, and D30-Cell + Cfs groups (*p* < 0.05). In contrast, D30-Cell (2.03 ± 0.56) and D30-Cell + Cfs (2.00 ± 0.40) showed the lowest FCR, significantly lower than D30-Control (2.78 ± 0.47) and D30-TSB (3.14 ± 0.30) (*p* < 0.05), while D30-Cfs (2.63 ± 0.66) was lower than D30-TSB (*p* < 0.05) but not different from D30-Control (*p* > 0.05). A similar trend was observed at Day 60, where D60-Cell (1.84 ± 0.35) and D60-Cell + Cfs (1.83 ± 0.33) maintained the lowest FCR, significantly lower than D60-Control (2.78 ± 0.47) and D60-TSB (2.91 ± 0.33) (*p* < 0.05), whereas D60-Cfs (2.64 ± 0.61) showed no difference from either control group (*p* > 0.05).

#### 3.2.2. Expression of Growth-Related Genes

At Day 30, *gh* expression in the brain was increased only in the Cell + Cfs group compared with D30-TSB (*p* < 0.05), but did not differ from D30-Control (*p* > 0.05). In the liver and muscle, *gh* was significantly upregulated in both Cell and Cell + Cfs compared with controls (*p* < 0.05), whereas the Cfs group showed no difference (*p* > 0.05). At Day 60, *gh* expression in the brain remained elevated in Cell and Cell + Cfs relative to both controls (*p* < 0.05). In the liver, *gh* expression in Cell and Cell + Cfs was higher than in D30-Control (*p* < 0.05) but not significantly different from D30-TSB (*p* > 0.05). In muscle, *gh* remained significantly higher in Cell and Cell + Cfs than in both controls (*p* < 0.05), while Cfs remained comparable (*p* > 0.05; Figure 2D–F).

### 3.3. Determining the Antioxidative Status and Oxidative Stress of Target Organs

#### 3.3.1. Spleen

**Figure 3:**
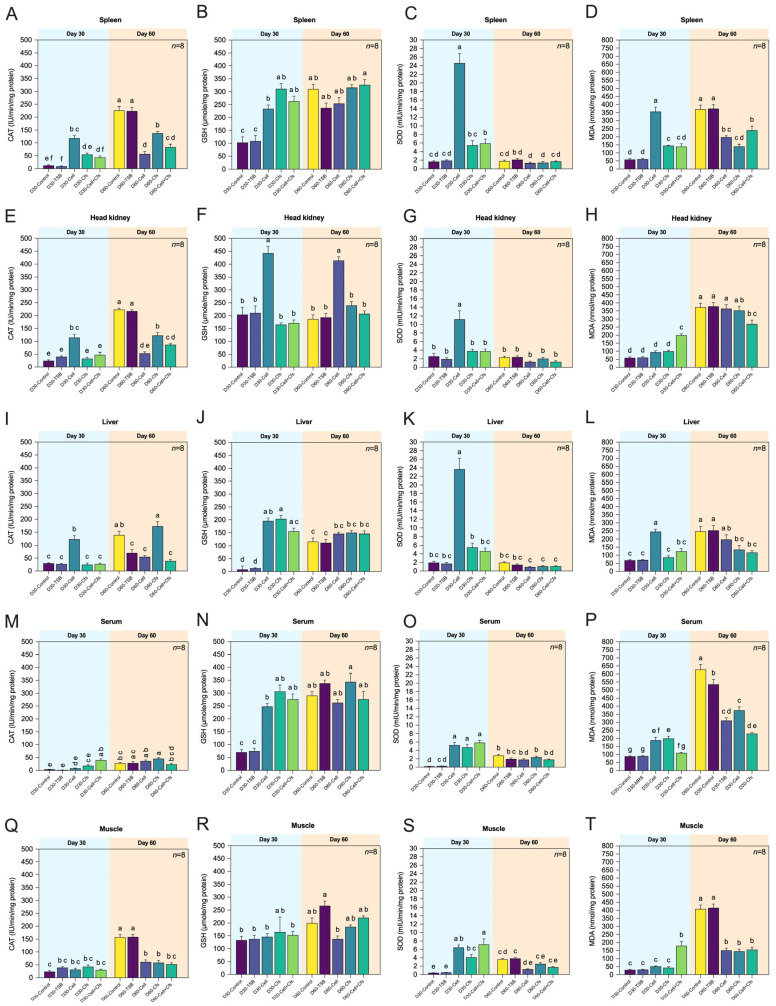
Antioxidant enzyme activities including catalase (CAT), glutathione (GSH), and superoxide dismutase (SOD)and levels of malondialdehyde (MDA) in spleen (**A**–**D**), head kidney (**E**–**H**), liver (**I**–**L**), serum (**M**–**P**), and muscle (**Q**–**T**) of tilapia following dietary supplementation with *B. velezensis* AAHM-BV2302 after 30 days of probiotic administration (Day 30) and 30 days post-supplementation (Day 60). Data are represented as mean ± SD (*n* = 8). Different letters above bars indicate statistically significant differences among groups (*p* < 0.05).

Overall, antioxidant enzyme activities in the spleen increased markedly during supplementation (Day 30) and decreased toward baseline after withdrawal (Day 60). The GSH level in the spleen showed a significant increase in the treatment groups at Day 30 compared with the controls (*p* < 0.05). The D30-Cell group exhibited the highest SOD activity among all groups, followed by D30-Cell + Cfs, and both were significantly higher than the two controls (*p* < 0.05). No significant difference was observed in D30-Cfs compared with the controls (*p* > 0.05). CAT activity was highest in D30-Cell, followed by D30-Cfs, which was significantly higher than D30-TSB (*p* < 0.05) but not different from D30-Control (*p* > 0.05). In contrast, D30-Cell + Cfs showed no significant difference from either control group (*p* > 0.05). By Day 60, CAT activity in both controls exceeded that of all treatment groups (*p* < 0.05). No significant differences in GSH levels or SOD activity were detected among the treatment groups compared with controls (*p* > 0.05). For MDA levels, significant reductions were observed in the Cell and Cfs groups at Day 30 (*p* < 0.05), while the Cell + Cfs group showed no significant difference compared with the controls (*p* > 0.05). At Day 60, however, both controls exhibited the highest MDA levels compared with all treatment groups (*p* < 0.05; Figure 3A–D).

#### 3.3.2. Head Kidney

In the head kidney, antioxidant responses were moderate and short-lived, with increased CAT, SOD, and GSH activities during supplementation (Day 30) and a general decline after withdrawal (Day 60). The activities of CAT, SOD, and GSH levels increased significantly only in the D30-Cell group at Day 30 compared with the controls (*p* < 0.05). No significant differences were observed in D30-Cfs and D30-Cell + Cfs relative to the controls (*p* > 0.05). By Day 60, CAT activity in both controls was significantly higher than among treatment groups (*p* < 0.05), whereas GSH levels remained higher in D60-Cell compared with the controls (*p* < 0.05). For SOD activity, no significant differences were detected between treatments and both controls (*p* > 0.05). The MDA levels were highest in Cell + Cfs at Day 30 compared with the other treatments and controls (*p* < 0.05), while Cell and Cfs did not differ from the controls (*p* > 0.05). By Day 60, MDA levels increased in both controls as well as in Cell and Cfs, all of which were significantly higher than in D60-Cell + Cfs (*p* < 0.05). No significant differences were detected for Cell and Cfs compared with the controls (*p* > 0.05; Figure 3E–H).

#### 3.3.3. Liver

Liver antioxidant enzymes showed tissue-specific fluctuations, with significant rises in CAT and SOD at Day 30 and reduced MDA levels in treated groups compared with controls. The CAT and SOD activities increased significantly only in the D30-Cell group at Day 30 compared with the controls (*p* < 0.05). No significant differences were observed in D30-Cfs and D30-Cell + Cfs relative to the controls (*p* > 0.05). For GSH levels, all treatment groups were generally higher than the D30-Control, although D30-Cfs was significantly lower than D30-TSB (*p* < 0.05). By Day 60, CAT activity was highest in D60-Cfs compared with D60-TSB, whereas D60-Control exceeded both D60-Cell and D60-Cell + Cfs (*p* < 0.05). No significant differences in GSH levels or SOD activity were detected among treatments and controls (*p* > 0.05). At Day 30, MDA levels were significantly higher in the Cell group than in all other treatments and controls (*p* < 0.05), whereas Cfs and Cell + Cfs did not differ from controls (*p* > 0.05). By Day 60, MDA levels in both controls were significantly higher than in D60-Cfs and D60-Cell + Cfs (*p* < 0.05), while D60-Cell remained comparable to controls (*p* > 0.05; Figure 3I–L).

#### 3.3.4. Serum

Serum antioxidant parameters reflected systemic redox improvement, as SOD, CAT, and GSH were elevated during supplementation and MDA levels remained lower in probiotic-fed fish throughout the experiment. The results showed that all treatment groups at Day 30 had higher levels of GSH and SOD activity compared with both controls (*p* < 0.05). A significant difference in CAT activity was observed only in D30-Cell + Cfs compared with the controls (*p* < 0.05), whereas D30-Cell and D30-Cfs showed no significant difference from controls (*p* > 0.05). By Day 60, CAT activity was significantly higher only in D60-Cfs compared with D60-Control (*p* < 0.05), while the other treatments showed no differences from either control (*p* > 0.05). Interestingly, GSH levels in both controls increased from Day 30, resulting in no significant differences among controls and treatments at Day 60 (*p* > 0.05). Likewise, no significant differences in SOD activity were detected between treatments and controls (*p* > 0.05). For MDA levels, significantly lower levels were observed in the Cell and Cfs groups at Day 30 (*p* < 0.05), whereas Cell + Cfs did not differ from the controls (*p* > 0.05). By Day 60, both controls exhibited the highest MDA levels compared with all treatment groups (*p* < 0.05; Figure 3M–P).

#### 3.3.5. Muscle

Overall, antioxidant activity in muscle showed only minor variations, with a transient increase in SOD activity at Day 30 and minimal changes in CAT, GSH, and MDA among treatments. The SOD activity among treatment groups was significantly higher than in both controls (*p* < 0.05), while CAT activity and GSH levels showed no significant differences between treatments and controls (*p* > 0.05). At Day 60, CAT activity increased in both controls compared with Day 30 and was significantly higher than in all treatment groups (*p* < 0.05). For GSH and SOD, only D60-Cell showed significantly lower levels than D60-TSB (*p* < 0.05), whereas D60-Cfs and D60-Cell + Cfs did not differ from either control (*p* > 0.05). At Day 30, MDA levels were significantly higher in Cell + Cfs compared with all other treatments and controls (*p* < 0.05), while Cell and Cfs did not differ from controls (*p* > 0.05). By Day 60, both controls exhibited the highest MDA levels compared with all treatment groups (*p* < 0.05; Figure 3Q–T).

### 3.4. Assessment of the Antioxidative Status and Oxidative Stress of Organs Associated with Mucosal Immunity

#### 3.4.1. Skin

**Figure 4:**
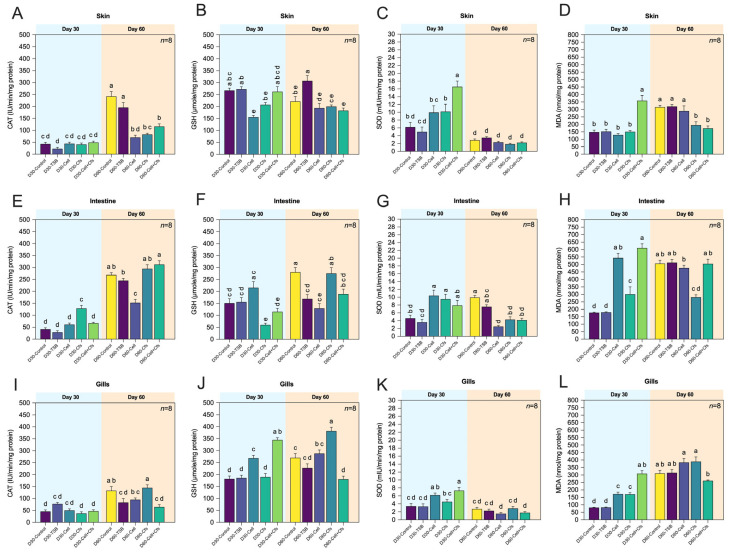
Antioxidant enzyme activities and the levels of malondialdehyde (MDA) in mucosal tissues of tilapia following dietary supplementation with *B*. *velezensis* AAHM-BV2302. Tissues include skin (**A**–**D**), intestine (**E**–**H**) and gills (**I**–**L**) after 30 days of probiotic administration (Day 30) and 30 days post-supplementation (Day 60). Data are represented as mean ± SD (*n* = 8). Different letters above bars indicate statistically significant differences among groups (*p* < 0.05).

In skin tissue, SOD and CAT activities increased markedly during supplementation (Day 30), whereas MDA levels declined, indicating enhanced local antioxidant status that gradually returned toward control values by Day 60. The activity of SOD was highest in the Cell + Cfs group at Day 30 compared with all other groups, and D30-Cfs also showed significantly higher activity than D30-TSB (*p* < 0.05). No significant difference was observed in D30-Cell compared with either control (*p* > 0.05). For GSH, only D30-Cell showed significantly lower levels than both controls (*p* < 0.05), while no significant differences were observed in the other treatment groups relative to the controls (*p* > 0.05). CAT activity did not differ significantly among groups (*p* > 0.05). By Day 60, both controls displayed the highest CAT activity, significantly exceeding that of all treatment groups (*p* < 0.05). A significant increase in GSH was also observed in D60-TSB compared with the other groups (*p* < 0.05). No significant differences were detected in any treatment groups compared with D60-Control (*p* > 0.05). Notably, SOD activity in all treatment groups decreased to levels comparable with both controls (*p* > 0.05). For MDA, levels were highest in Cell + Cfs at Day 30 compared with all other treatments and controls (*p* < 0.05), while D30-Cell and D30-Cfs did not differ from controls (*p* > 0.05). By Day 60, both controls exhibited the highest MDA levels compared with Cfs and Cell + Cfs (*p* < 0.05), whereas MDA levels in the Cell group remained comparable to controls (*p* > 0.05; Figure 4A–D).

#### 3.4.2. Intestine

Antioxidant responses in the intestine varied among treatments, with pronounced peaks in CAT and SOD at Day 30 and a normalization trend after withdrawal (Day 60). MDA levels remained lower in all treated groups compared with controls. At Day 30 after probiotic supplementation, CAT activity was highest in the D30-Cfs group among all treatments (*p* < 0.05), while the other treatment groups did not differ from the two controls (*p* > 0.05). In contrast, GSH levels were lowest in D30-Cfs, whereas D30-Cell and D30-Cell + Cfs did not differ significantly from the controls (*p* > 0.05). For SOD activity, both D30-Cell and D30-Cfs were significantly higher than the controls (*p* < 0.05). Notably, D30-Cell + Cfs was significantly higher than D30-TSB (*p* < 0.05) but did not differ from D30-Control (*p* > 0.05). By Day 60, CAT activity was highest in Cell + Cfs compared with D60-TSB, while the lowest CAT activity was observed in the Cell group relative to both controls (*p* < 0.05). No significant differences were detected between D60-Cfs and the controls (*p*> 0.05). GSH levels were highest in D60-Control compared with Cell, Cell + Cfs, and D60-TSB, whereas D60-Cfs was significantly higher than D60-TSB (*p* < 0.05) but not different from D60-Control (*p* > 0.05). The SOD activity in all treatment groups was significantly lower than in D60-Control (*p* < 0.05). D60-TSB exhibited higher SOD activity than D60-Cell (*p* < 0.05), whereas no significant differences were observed for D60-Cfs and D60-Cell + Cfs relative to controls (*p* > 0.05). At Day 30, all treatment groups exhibited higher MDA levels than both controls (*p* < 0.05). By Day 60, MDA levels in the controls increased to levels comparable to those in D60-Cell and D60-Cell + Cfs (*p* > 0.05), whereas D60-Cfs showed the lowest MDA level compared with both controls and the other treatments (*p* < 0.05; Figure 4E–H).

#### 3.4.3. Gills

Gill tissues exhibited notable antioxidant activation during supplementation, as GSH and SOD activities were elevated and MDA concentrations reduced in Cell and Cell + Cfs groups, particularly at Day 30. These effects were partly retained after withdrawal. For Day 30, GSH levels and SOD activity in D30-Cell and D30-Cell + Cfs were significantly higher than in both controls (*p* < 0.05), whereas the Cfs group did not differ significantly from the controls (*p* > 0.05). CAT activity showed no significant differences between treatments and controls (*p* > 0.05). By Day 60, CAT activity and GSH levels in D60-Cell + Cfs were lower than in D60-Control (*p* < 0.05) but did not differ from D60-TSB (*p* > 0.05). Additionally, D60-Cfs showed significantly higher GSH levels than both controls (*p* < 0.05) and higher CAT activity than D60-TSB, but not different from D60-Control (*p* > 0.05). No significant differences in SOD activity were observed among treatments relative to both controls (*p* > 0.05). At Day 30, all treatment groups exhibited significantly higher MDA levels compared with both controls (*p* < 0.05). By Day 60, however, MDA levels were lowest in D60-Cell + Cfs compared with both controls and the other treatment groups (*p* < 0.05), while the two controls had increased to levels comparable to those in D60-Cell and D60-Cfs (*p* > 0.05; Figure 4I–L).

### 3.5. Effects of Bacillus velezensis AAHM-BV2302 on Humoral Immune Responses and IgM of Tilapia

#### 3.5.1. Lysozyme Activity

**Figure 5:**
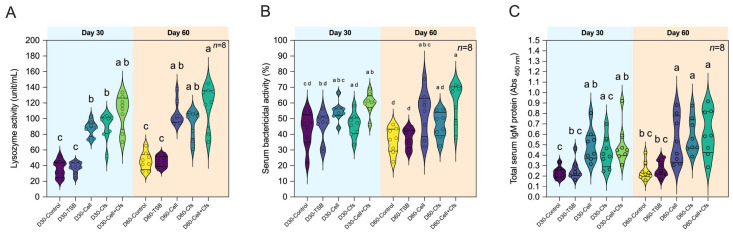
Humoral immune responses of tilapia following dietary supplementation with *B*. *velezensis* AAHM-BV2302. Parameters include lysozyme activity (**A**), serum bactericidal activity (**B**) and total serum IgM levels (**C**) after 30 days of probiotic administration (Day 30) and 30 days post-supplementation (Day 60). Data are represented as mean ± SD (*n* = 8). Different letters above bars indicate statistically significant differences among groups (*p* < 0.05).

The lysozyme activity in serum increased markedly in all treatment groups at Day 30, with D30-Cell + Cfs showing the highest value with 108.42 ± 22.47 unit/mL, followed by D30-Cell and D30-Cfs with 87.69 ± 9.81 and 90.19 ± 17.72 unit/mL, respectively. All treatment groups exhibited significantly higher activity than the two controls (*p* < 0.05). At Day 60, lysozyme activity remained elevated in all treatment groups compared with both controls (*p* < 0.05; Figure 5A).

#### 3.5.2. Bactericidal Activity

At Day 30, bactericidal activity against Gram-positive *S. agalactiae* was significantly higher only in the Cell + Cfs group compared with D30-Control, with a value of 59.93 ± 6.77% (*p* < 0.05), but it did not differ from D30-TSB (*p* > 0.05). No significant differences were observed in the Cell and Cfs groups relative to the controls (*p* > 0.05). Interestingly, at Day 60, bactericidal activity in both Cell and Cell + Cfs remained significantly higher than in the controls despite the withdrawal of probiotic feeding, indicating a sustained protective effect. The values were 53.41 ± 14.54% and 61.26 ± 12.57%, respectively (*p* < 0.05). In contrast, D60-Cfs did not differ significantly from the controls (*p* > 0.05; Figure 5B).

#### 3.5.3. Total Serum IgM

Total serum IgM levels increased significantly at Day 30 in the D30-Cell and D30-Cell + Cfs groups, with absorbance values of 0.50 ± 0.16 and 0.50 ± 0.19, respectively, compared with D30-Control (0.23 ± 0.05) and D30-TSB (0.26 ± 0.09) (*p* < 0.05). No significant difference was observed in the D30-Cfs group compared with either control (*p* > 0.05). At Day 60, IgM levels remained significantly higher in D60-Cell, D60-Cell + Cfs, and notably also in D60-Cfs, compared with both controls (*p* < 0.05; Figure 5C).

### 3.6. Effects of Probiotic Supplementation on the Expression of Immune-Related Genes

**Figure 6:**
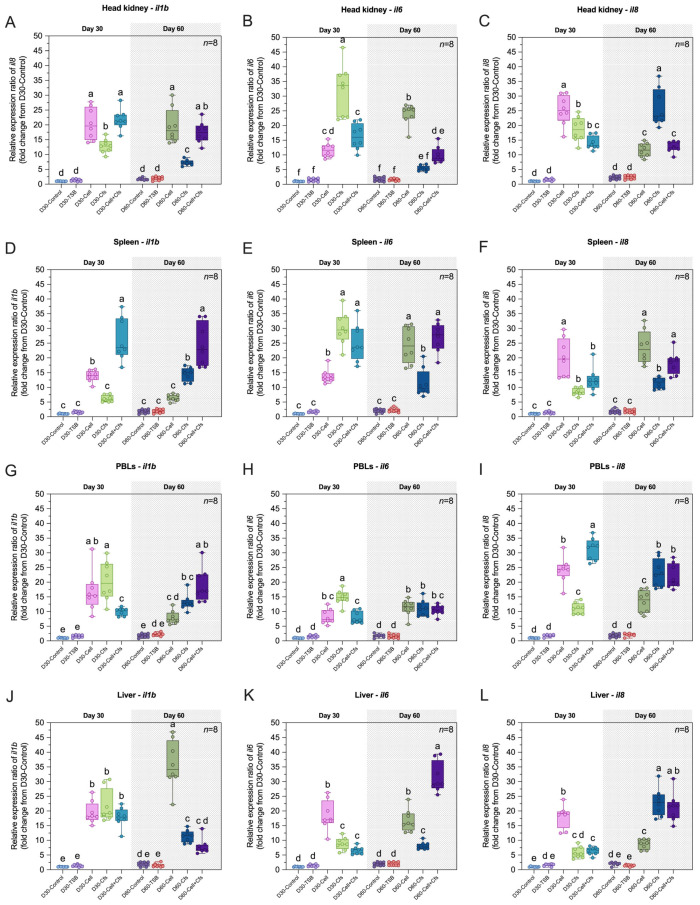

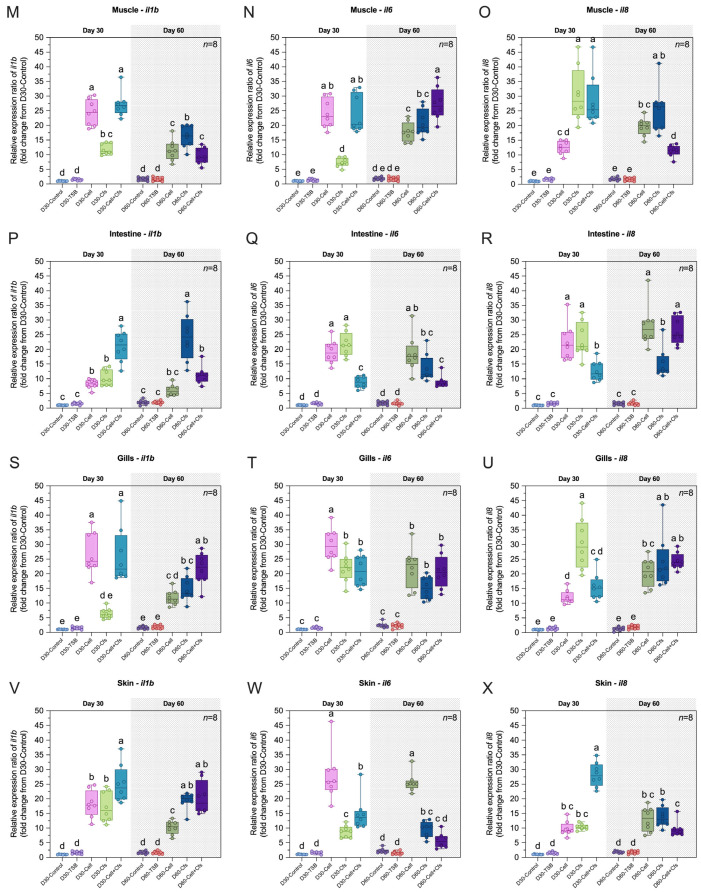
Relative expression (fold change) of immune-related genes including *il1b*, *il6* and *il8* in head kidney (**A**–**C**), spleen (**D**–**F**), PBLs (**G**–**I**), liver (**J**–**L**), muscle (**M**–**O**), intestine (**P**–**R**), gills (**S**–**U**) and skin (**V**–**X**) of tilapia following dietary supplementation with *B*. *velezensis* AAHM-BV2302 after 30 days of probiotic administration (Day 30) and 30 days post-supplementation (Day 60). Data are represented as mean ± SD (*n* = 8). Different letters above bars indicate statistically significant differences among groups (*p* < 0.05).

The expression levels of *il1b*, *il6* and *il8* in the head kidney, spleen, PBLs, liver, muscle, intestine, gills and skin are presented in Figure 6A–X.

#### 3.6.1. Expression of *il1b* Gene

The expression of *il1b* was strongly induced by probiotic supplementation at both Day 30 and Day 60. At Day 30, *il1b* increased significantly in all treatment groups in the head kidney, PBLs, liver, muscle, intestine, and skin compared with the controls (*p* < 0.05). In the spleen and gills, significant upregulation was observed only in the Cell and Cell + Cfs groups compared with both controls (*p* < 0.05), whereas D30-Cfs did not differ from the controls (*p* > 0.05). At Day 60, *il1b* remained significantly upregulated in the head kidney, muscle, gills, and skin compared with the two control groups (*p* < 0.05). In the spleen and intestine, significant increases were observed in D60-Cfs and D60-Cell + Cfs compared with both controls (*p* < 0.05), while D60-Cell did not differ from controls (*p* > 0.05). In PBLs, significant upregulation was observed in the Cfs and Cell + Cfs groups compared with both controls (*p* < 0.05), and additionally the D60-Cell group showed significant upregulation compared with D60-Control (*p* < 0.05) but not with D60-TSB (*p* > 0.05). In the liver, D60-Cell and D60-Cfs were significantly upregulated relative to both controls (*p* < 0.05), whereas Cell + Cfs showed significant upregulation compared with D60-TSB (*p* < 0.05) but did not differ from D60-Control (*p* > 0.05; Figure 6A,D,G,J,M,P,S,V).

#### 3.6.2. Expression of *il6* Gene

The expression of *il6* was strongly induced by probiotic supplementation at both Day 30 and Day 60. At Day 30, *il6* increased significantly in all treatment groups in the head kidney, spleen, PBLs, liver, muscle, intestine, gills, and skin compared with the controls (*p* < 0.05). At Day 60, *il6* remained significantly upregulated in the spleen, PBLs, liver, muscle, intestine, gills, and skin compared with the controls (*p* < 0.05). In the head kidney, significant upregulation was observed in the Cell and Cell + Cfs groups compared with both controls (*p* < 0.05), whereas no significant difference was detected in the Cfs group compared with either control (*p* > 0.05; Figure 6B,E,H,K,N,Q,T,W).

#### 3.6.3. Expression of *il8* Gene

The expression of *il8* was strongly induced by probiotic supplementation at both Day 30 and Day 60. At Day 30, *il8* increased significantly in all treatment groups in the head kidney, spleen, PBLs, muscle, intestine, gills, and skin compared with the controls (*p* < 0.05). In the liver, *il8* was upregulated in the Cell and Cell + Cfs groups compared with both controls (*p* < 0.05), while D30-Cfs showed a significant increase only relative to D30-Control (*p* < 0.05) but not to D30-TSB (*p* > 0.05). By Day 60, *il8* remained significantly upregulated across all treatment groups in these tissues compared with both controls (*p* < 0.05; Figure 6C,F,I,L,O,R,U,X).

### 3.7. Challenge Test

**Figure 7:**
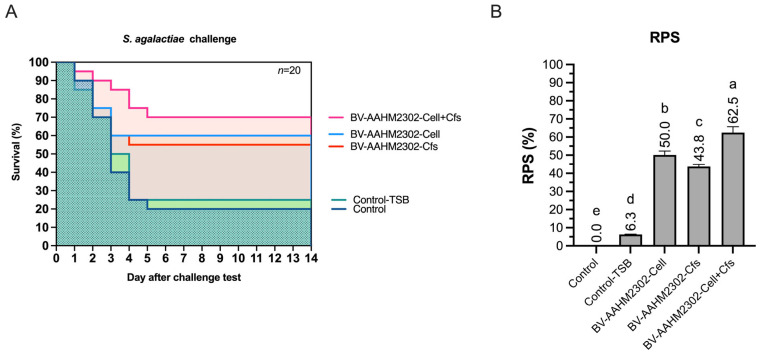
Survival probability (**A**) and relative percent survival (RPS) (**B**) of tilapia were determined following *S. agalactiae* challenge at day 60, after an additional 30 days of dietary supplementation with *B*. *velezensis* AAHM-BV2302. Kaplan–Meier survival curves are shown for D60-Control, D60-TSB, D60-Cell, D60-Cfs, and D60-Cell + Cfs groups (*n* = 20). Different letters above bars indicate statistically significant differences among groups (*p* < 0.05).

The survival rate (SR) following *S. agalactiae* challenge differed significantly among treatment groups (*p* < 0.05). The D60-Cell + Cfs group achieved the highest SR (70%) with a relative percent survival (RPS) of 62.5%, indicating a strong protective effect against the pathogen. The D60-Cell and D60-Cfs groups followed with SR values of 60% and 55% and RPS values of 50% and 43.8%, respectively. All treatment groups exhibited significantly higher survival than the controls, where SR values were 20% (D60-Control) and 25% (D60-TSB) with an RPS of only 6.3% (*p* < 0.05) (Figure 7A,B).

## 4. Discussion

The present study provides an integrative evaluation of the probiotic, postbiotic (Cfs) and combination between probiotic and postbiotic (Cell + Cfs) potential of *B. velezensis* AAHM-BV2302 in tilapia, encompassing antioxidative capacity, mucosal barrier function, immune modulation, and resistance to *S. agalactiae*. Whole-genome sequencing and phylogenomic comparisons confirmed its taxonomic placement within *B. velezensis* (genome size 4,156,630 bp; GC 45.9%; 4103 CDSs; ANI 99.43% and dDDH 97.3% to *B. velezensis* NRRL B-41580; ANI 98.04% and dDDH 90.6% to *B. amyloliquefaciens* subsp. *plantarum* FZB42), thereby validating strain identity without inferring functional gene content beyond assembly and similarity metrics reported in the results. Reported genomes of *B. velezensis* strains generally range between 3.9–4.3 Mb with GC contents of 45.8–46.7%. The genome of *B. velezensis* GY65 isolated from mandarin fish (*Siniperca chuatsi*) consisted of a circular chromosome of 3,929,765 bp with a GC content of 46.50% [50]. The genome of *B. velezensis* CPA1-1 comprised a 3,925,520 bp circular chromosome with an average GC content of 46.49%, including 3762 protein-coding genes [51]. In addition, the genome of *B. velezensis* VJH504 consisted of a circular chromosome of 3,980,733 bp with a GC content of 46.46% [52]. Thus, the genomic validation of AAHM-BV2302 as *B. velezensis*, together with its host-adapted origin from the tilapia intestine and consistent functional performance, supports the view that host-adapted *B. velezensis* strains are particularly well-suited to deliver broad-spectrum benefits in aquaculture. These findings emphasize the value of coupling genomic identification with functional assessment to establish reliable, strain-specific probiotic strategies.

The present study demonstrated clear growth promotion by *B*. *velezensis* AAHM-BV2302, as ADG and TWG were significantly higher in the Cell and Cell + Cfs groups at both Day 30 and Day 60, while FCR improved transiently at Day 30 in Cell + Cfs, indicating a rapid onset of benefits with partial persistence after post-supplementation. This accelerated effect may be attributed to the spore-forming capacity of *B. velezensis*, allowing rapid gut colonization and secretion of extracellular enzymes that enhance nutrient utilization and digestive efficiency [18]. Such growth-promoting effects are consistent with previous studies on freshwater species such as striped catfish [53], Nile tilapia [23,54], and Amur minnow [55], where *Bacillus* supplementation improved weight gain and FCR. In addition, the upregulation of the growth hormone (*gh*) gene in the brain, liver, and muscle further supports the observed growth enhancement, suggesting activation of the somatotropic axis and increased anabolic activity. Similar findings have been reported in Nile tilapia fed with multi-strains *Bacillus* spp., where *gh* and *igf1* expression were elevated alongside improved feed conversion and muscle development [56]. In yellow perch (*Perca flavescens*), where *Bacillus*-supplementation enhanced *gh* and *igf1* transcription alongside improved growth performance [57], reinforcing that probiotic *Bacillus* strains can modulate endocrine growth regulation in fish.

In this study, activities of major antioxidant enzymes including SOD, CAT, and GSH increased significantly in several tissues at Day 30, while MDA levels were markedly reduced compared with the controls. By Day 60, CAT activity partially persisted in the spleen, serum, and head kidney, whereas SOD and GSH trended back toward control levels, and MDA remained lower in Cfs and Cell + Cfs groups, particularly in the spleen, gills, intestine, and skin. These findings indicate that *B. velezensis* AAHM-BV2302 supplementation effectively enhanced redox homeostasis and mitigated oxidative stress during both active feeding and the withdrawal phase. Major antioxidant enzymes, including SOD, GSH, and CAT, constitute the first line of defense against reactive oxygen species (ROS) in fish [58]. SOD catalyzes the dismutation of superoxide anions into hydrogen peroxide, which is subsequently decomposed by CAT, while GSH serves as a versatile thiol buffer to neutralize secondary radicals and maintain redox balance [59,60]. Elevated activities of these enzymes generally denote enhanced antioxidant capacity, while a decline in MDA, the principal biomarker of lipid peroxidation [61], indicates reduced ROS-driven cellular injury [62]. Probiotics such as *Bacillus* spp. can stimulate host antioxidant systems through both antigenic stimulation and bioactive metabolites, including organic acids and lipopeptides, which enhance enzymatic defense and attenuate free radical–induced damage [63]. Previous studies on *Bacillus* supplementation in fish have reported comparable antioxidant responses. Elbahnaswy et al. [64] demonstrated that tilapia fed diets containing mixed *Bacillus* strains showed significant increases in SOD and CAT activities and concomitant reductions in MDA, both under basal conditions and following a heat challenge. Similarly, Yu et al. [23] found that dietary *B. subtilis* in Nile tilapia markedly enhanced SOD and CAT activities while lowering MDA levels. In mandarin fish, *B*. *velezensis* GY65 supplementation also elevated serum SOD and CAT [50]. Furthermore, studies on mixed *Bacillus* spp. revealed a strong negative correlation between antioxidant enzyme activities of CAT, SOD and MDA concentrations in European seabass [65]. Collectively, these findings suggest that AAHM-BV2302 enhances oxidative balance in tilapia through dual actions-enzymatic redox buffering during feeding and metabolite-mediated protection thereafter.

Innate humoral immunity represents the first line of systemic defense in teleost and includes soluble effectors such as lysozyme, the complement system, and natural IgM antibodies, all of which play central roles in limiting early pathogen proliferation [66]. Lysozyme hydrolyzes bacterial peptidoglycan and destabilizes cell walls, thereby exerting rapid, broad-spectrum antimicrobial action [67], while serum bactericidal activity reflects the combined action of lysozyme, complement, and antimicrobial metabolites [66]. IgM, the predominant antibody isotype in fish, provides natural antibody activity, fixes complement, and bridges innate and adaptive immunity, making it a critical indicator of humoral readiness [68]. In the present study, lysozyme activity increased significantly in all treatment groups at Day 30 and remained elevated at Day 60, while bactericidal activity persisted in the Cell and Cell + Cfs groups post probiotic supplementation, and total serum IgM rose early (Cell and Cell + Cfs at Day 30) and was maintained across all treatment groups by Day 60, indicating sustained humoral priming beyond the feeding phase. Similar enhancements of lysozyme activity, serum bactericidal activity, and total IgM following dietary *Bacillus* supplementation have also been reported in several aquatic animal species, supporting the conserved capacity of *Bacillus* probiotics to strengthen innate humoral immunity [69,70,71,72]. The magnitude and persistence of these humoral responses in our study, particularly under the Cell + Cfs treatment, highlight the strong immunostimulatory efficacy of host-adapted *B. velezensis* AAHM-BV2302, reinforcing its potential as a robust probiotic candidate for tilapia health management.

IL-1β, IL-6, and IL-8 are key pro-inflammatory cytokines that regulate immune activation and host defense [73]. IL-1β initiates inflammation, recruits leukocytes, and modulates coagulation pathways [74]. IL-6 drives the acute-phase response, promotes B-cell differentiation, and links immunity with coagulation through tissue factor regulation [75]. IL-8 functions mainly as a chemokine for neutrophil recruitment and activation, while also contributing to platelet activation [76]. Together, these cytokines coordinate leukocyte mobilization, inflammation, and hemostatic regulation, forming a critical axis of innate immune protection [77]. In the present study, all three cytokines were markedly upregulated in hematopoietic and mucosal tissues at Day 30, with many responses persisting to Day 60. This sustained transcriptional priming paralleled elevated lysozyme and bactericidal activities together with reduced MDA, indicating a balanced cytokine environment that facilitates pathogen clearance while limiting oxidative damage. The concerted induction across systemic organs (spleen, head kidney, serum) and barrier sites (gill, skin, intestine) suggests that *B. velezensis* AAHM-BV2302 enhances multilayer host defense at both first-contact and central immune compartments. Similar patterns have been described in fish receiving probiotic *Bacillus*, where increased expression of *il1b*, *il6*, *il8*, or broader cytokine panels has been linked to improved disease resistance, supporting the robust Cell and Cell + Cfs responses and their persistence beyond the supplementation period. Comparable outcomes have been reported in red seabream, where dietary mixture probiotic supplement significantly elevated *il1b* expression [78] and supplementation with *Bacillus* sp. PM8313 further upregulated *il6* expression [79]. Likewise, *B. velezensis* supplementation in mandarin fish was shown to upregulate *il8* transcripts [50]. These consistent findings reinforce the capacity of *Bacillus* probiotics to modulate pro-inflammatory cytokine axes in fish, supporting the durable Cell and Cell + Cfs responses observed in our study.

In the present study, survival following *S. agalactiae* challenge was markedly improved in all probiotic-fed groups. The Cell + Cfs treatment achieved the highest survival rate of 70% with an RPS of 62.5, followed by Cell and Cfs, each significantly outperforming the controls. These findings demonstrate that AAHM-BV2302 supplementation provided strong protection under pathogen stress. Live *Bacillus* spores can persist in the gastrointestinal tract, establish colonization resistance, and stimulate host immune receptors to sustain humoral responses such as lysozyme activity, bactericidal activity, and total IgM. At the same time, postbiotic metabolites including lipopeptides and organic acids exert direct antimicrobial effects and modulate inflammation, thereby reducing oxidative damage and enhancing clearance of invading bacteria. The superior protection in the Cell + Cfs treatment reflects the synergy of these complementary actions. Similar improvements in survival have been reported in other aquaculture species supplemented with *Bacillus*. For example, dietary *B. velezensis* FLU-1 significantly increased survival of largemouth bass challenged with *Aeromonas hydrophila* by reducing pathogen load in liver tissue [80]. In zebrafish, fish-derived *B. velezensis* strains enhanced protection against *Aeromonas veronii*, with survival rates markedly higher than controls [81]. Likewise, supplementation with *B. amyloliquefaciens* improved survival of Nile tilapia exposed to *A. hydrophila*, reaching over 90% survival compared with sharply lower rates in controls [82]. Collectively, these findings, together with our present results, confirm that *Bacillus* probiotics confer broad protective benefits against diverse bacterial pathogens in fish, with synergistic formulations such as Cell + Cfs offering particularly robust outcomes.

## 5. Conclusions

Whole-genome sequencing confirmed the taxonomic identity and safety of *Bacillus velezensis* AAHM-BV2302, without direct inference of functional genes. This study demonstrates that dietary supplementation with *B. velezensis* AAHM-BV2302—as cells (Cell), cell-free supernatant (Cfs), or their combination (Cell + Cfs)—enhanced growth performances, improved antioxidant capacity across multiple tissues, boosted lysozyme and bactericidal activities with partially sustained effects after withdrawal, elevated serum IgM, broadly upregulated pro-inflammatory cytokines across systemic and mucosal compartments, and conferred substantial protection against *S*. *agalactiae*. The Cell + Cfs regimen consistently exhibited the strongest overall effects, supporting a probiotic–postbiotic synergy model. These outcomes, while varying among tissues, indicate that the persistence of antioxidant and immune responses depends on tissue-specific redox and immune dynamics. Additionally, these findings position *B. velezensis* AAHM-BV2302 as a safe, multifunctional, and scalable probiotic–postbiotic candidate for sustainable tilapia aquaculture, with practical potential to reduce disease losses and antibiotic dependence under intensive rearing conditions. Future studies should further clarify metabolite-mediated mechanisms, gut colonization dynamics, and long-term persistence to optimize its application.

## Figures and Tables

**Table 1:** Primers used in the qRT-PCR analysis of gene expression.

Gene Group	Genes	Nucleotide Sequences (5′→3′)	Tm (°C)	Efficiency (%)	Reference
Reference gene	*Actin*, *beta 1* (*actb1*)	F: ACAGGATGCAGAAGGAGATCACAG R: GTACTCCTGCTTGCTGATCCACAT	60	101.6	[38]
	18S rRNA (*rna18s*)	F: GGACACGGAAAGGATTGACAG R: GTTCGTTATCGGAATTAACCAGAC	60	98.9	[12]
Growth-related gene	Growth hormone (*gh*)	F: TTACATCATCAGCCCGATCG R: AGATCGACAGCAGCTTCAGGA	60	95.9	[47]
Cytokines and signaling molecules	Interleukin-1β (*il1b*)	F: GTGCTGAGCACAGAATTCCAGGAT R: GAAGAACCAAGCTCCTCTTTTGGC	60	100.3	[48]
	Interleukin-6 (*il6*)	F: ACAGAGGAGGCGGAGATG R: GCAGTGCTTCGGGATAGAG	60	98.7	[38]
	Interleukin-8 (*il8*)	F: GCCTCTTCAGGGCTAGAGTCA R: TGAAGCCTGAAGCGCTAAACT	60	99.5	[38]

**Table 2:** Genome features of *Bacillus velezensis* AAHM-BV2302.

Organism	*Bacillus velezensis* AAHM-BV2302
BioProject no.	PRJNA1320929
Accession no.	JBQXGX000000000
Size	4,156,630 bp
GC content	45.9%
CDS	4103
Gene	4177
tRNA	69
rRNA	4
tmRNA	1

**Table 3:** Taxonomic identification of *Bacillus velezensis* AAHM-BV2302 based on Average Nucleotide Identity (ANI), digital DNA–DNA hybridization (dDDH), and nucleotide similarity compared with reference genomes.

Reference Genome	ANI	dDDH (d6, in %)	Nucleotide Similarity (%)
*Bacillus velezensis* NRRL B-41580	99.43	97.3	93.42
*Bacillus amyloliquefaciens* subsp. *plantarum* FZB42	98.04	90.6	87.09

## Data Availability

The original contributions presented in this study are included in the article. Further inquiries can be directed to the corresponding author(s).

## References

[1] Wang M., Lu M. (2016). Tilapia polyculture: A global review. Aquac. Res..

[2] El-Sayed A.F.M., Fitzsimmons K. (2023). From Africa to the world—The journey of Nile tilapia. Rev. Aquac..

[3] Fimbres-Acedo Y.E., Maeda-Martínez A.N., Garza-Torres R. (2025). Tilapia diseases reported in Mexico: A systematic review. J. Fish Dis..

[4] Gundi V.A., Bogireddy D., Vundru A.K., Arthala P.K., Vadela M.B., Karri S., Allam U.S., Gujjula M.S., Kodali V.P. (2025). Microbial pathogens in aquaculture: A review of emerging threats. Acad. Biol..

[5] Sukkarun P., Kitiyodom S., Kamble M.T., Bunnoy A., Boonanuntanasarn S., Yata T., Boonrungsiman S., Thompson K.D., Rodkhum C., Pirarat N. (2024). Systemic and mucosal immune responses in red tilapia (*Oreochromis* sp.) following immersion vaccination with a chitosan polymer-based nanovaccine against *Aeromonas veronii*. Fish Shellfish Immunol..

[6] Abdallah E.S.H., Metwally W.G.M., Abdel-Rahman M.A.M., Albano M., Mahmoud M.M. (2024). *Streptococcus agalactiae* infection in Nile tilapia (*Oreochromis niloticus*): A review. Biology.

[7] Surachetpong W., Janetanakit T., Nonthabenjawan N., Tattiyapong P., Sirikanchana K., Amonsin A. (2017). Outbreaks of tilapia lake virus infection, Thailand, 2015–2016. Emerg. Infect. Dis..

[8] Elgendy M.Y., Sherif A.H., Kenawy A.M., Abdelsalam M. (2022). Phenotypic and molecular characterization of the causative agents of edwardsiellosis causing Nile tilapia (*Oreochromis niloticus*) summer mortalities. Microb. Pathog..

[9] Sherif A.H., Kassab A.S. (2023). Multidrug-resistant Aeromonas bacteria prevalence in Nile tilapia broodstock. BMC Microbiol..

[10] Poudyal S., Pulpipat T., Wang P.C., Chen S.C. (2020). Comparison of the pathogenicity of *Francisella orientalis* in Nile tilapia (*Oreochromis niloticus*), Asian seabass (*Lates calcarifer*) and largemouth bass (*Micropterus salmoides*) through experimental intraperitoneal infection. J. Fish Dis..

[11] Cortés-Sánchez A.D.J., Espinosa-Chaurand L.D., Díaz-Ramirez M., Torres-Ochoa E. (2021). *Plesiomonas*: A review on food safety, fish-borne diseases, and tilapia. Sci. World J..

[12] Thangsunan P., Thangsunan P., Mahatnirunkul T., Buncharoen W., Saenphet K., Saenphet S., Phaksopa J., Thompson K.D., Srisapoome P., Kumwan B. (2025). Development and characterization of an innovative *Flavobacterium oreochromis* antigen-encapsulated hydrogel bead for enhancing oral vaccine delivery in hybrid red tilapia (*Oreochromis* spp.). Fish Shellfish Immunol..

[13] Watts J.E.M., Schreier H.J., Lanska L., Hale M.S. (2017). The rising tide of antimicrobial resistance in aquaculture: Sources, sinks and solutions. Mar. Drugs.

[14] Yuan X., Lv Z., Zhang Z., Han Y., Liu Z., Zhang H. (2023). A review of antibiotics, antibiotic resistant bacteria, and resistance genes in aquaculture: Occurrence, contamination, and transmission. Toxics.

[15] Hoseinifar S.H., Yousefi S., Van Doan H., Ashouri G., Gioacchini G., Maradonna F., Carnevali O. (2020). Oxidative stress and antioxidant defense in fish: The implications of probiotic, prebiotic, and synbiotics. Rev. Fish Sci. Aquac..

[16] Ringø E., Harikrishnan R., Soltani M., Ghosh K. (2022). The effect of gut microbiota and probiotics on metabolism in fish and shrimp. Animals.

[17] Shehata A.I., Soliman A.A., Ahmed H.A., Gewaily M.S., Amer A.A., Shukry M., Abdel-Latif H.M.R. (2024). Evaluation of different probiotics on growth, body composition, antioxidant capacity, and histoarchitecture of *Mugil capito*. Sci. Rep..

[18] Elshaghabee F.M.F., Rokana N., Gulhane R.D., Sharma C., Panwar H. (2017). *Bacillus* as potential probiotics: Status, concerns, and future perspectives. Front. Microbiol..

[19] Nayak S.K. (2021). Multifaceted applications of probiotic *Bacillus* species in aquaculture with special reference to *Bacillus subtilis*. Rev. Aquac..

[20] Fazle Rabbee M., Baek K.H. (2020). Antimicrobial activities of lipopeptides and polyketides of *Bacillus velezensis* for agricultural applications. Molecules.

[21] Kaspar F., Neubauer P., Gimpel M. (2019). Bioactive secondary metabolites from *Bacillus subtilis*: A comprehensive review. J. Nat. Prod..

[22] Markelova N., Chumak A. (2025). Antimicrobial activity of *Bacillus* cyclic lipopeptides and their role in the host adaptive response to changes in environmental conditions. Int. J. Mol. Sci..

[23] Yu H., Nazir S., Ijaz F., Zahid M.U., Mushtaq M., Khan M., Rahman A., Rahman M.A.U. (2025). Dietary supplementation of *Bacillus subtilis* as probiotic influenced the growth performance, hematological parameters, immune function, antioxidant status, and digestive enzyme activity of Nile tilapia fingerlings (*Oreochromis niloticus*). Animals.

[24] Wiratama N., Kumwan B., Meachasompop P., Adisornprasert Y., Srisapoome P., Thompson K.D., Phrompanya P., Thangsunan P., Thangsunan P., Saenphet K. (2025). Probiotic and postbiotic effects of *Bacillus velezensis* AAHM-BV2354 on boosting immunity, growth performance, antioxidant activity and resistance to *Edwardsiella tarda* infection in pangasius (*Pangasianodon hypophthalmus*). Fish Shellfish Immunol..

[25] Wiratama N., Meachasompop P., Kumwan B., Adisornprasert Y., Srisapoome P., Phrompanya P., Thangsunan P., Thangsunan P., Saenphet K., Saenphet S. (2025). Dietary probiotic *Bacillus subtilis* AAHM-BS2360 and Its postbiotic metabolites enhance growth, immunity, and resistance to edwardsiellosis in *Pangasianodon hypophthalmus*. Antioxidants.

[26] Wu Z., Qi X., Qu S., Ling F., Wang G. (2021). Dietary supplementation of *Bacillus velezensis* B8 enhances immune response and resistance against *Aeromonas veronii* in grass carp. Fish Shellfish Immunol..

[27] Thorakkattu P., Khanashyam A.C., Shah K., Babu K.S., Mundanat A.S., Deliephan A., Deokar G.S., Santivarangkna C., Nirmal N.P. (2022). Postbiotics: Current trends in food and pharmaceutical industry. Foods.

[28] Tao L.-T., Lu H., Xiong J., Zhang L., Sun W.-W., Shan X.-F. (2024). The application and potential of postbiotics as sustainable feed additives in aquaculture. Aquaculture.

[29] Thakur K., Singh B., Kumar S., Sharma D., Sharma A.K., Jindal R., Kumar R. (2025). Potential of probiotics and postbiotics in aquaculture: Connecting current research gaps and future perspectives. Microbe.

[30] Bolger A.M., Lohse M., Usadel B. (2014). Trimmomatic: A flexible trimmer for Illumina sequence data. Bioinformatics.

[31] Prjibelski A., Antipov D., Meleshko D., Lapidus A., Korobeynikov A. (2020). Using SPAdes *de novo* assembler. Curr. Protoc. Bioinform..

[32] Seemann T. (2014). Prokka: Rapid prokaryotic genome annotation. Bioinformatics.

[33] Grant J.R., Enns E., Marinier E., Mandal A., Herman E.K., Chen C.-Y., Graham M., Van Domselaar G., Stothard P. (2023). Proksee: In-depth characterization and visualization of bacterial genomes. Nucleic Acids Res..

[34] Meier-Kolthoff J.P., Göker M. (2019). TYGS is an automated high-throughput platform for state-of-the-art genome-based taxonomy. Nat. Commun..

[35] Trifinopoulos J., Nguyen L.T., von Haeseler A., Minh B.Q. (2016). W-IQ-TREE: A fast online phylogenetic tool for maximum likelihood analysis. Nucleic Acids Res..

[36] Ott B.D., Torrans E.L., Tucker C.S. (2024). Fish production, water quality, and the role of nitrification as an ammonia removal process in intensively aerated hybrid catfish ponds. J. World Aquac. Soc..

[37] Meachasompop P., Bunnoy A., Keaswejjareansuk W., Dechbumroong P., Namdee K., Srisapoome P. (2024). Development of immersion and oral bivalent nanovaccines for streptococcosis and columnaris disease prevention in fry and fingerling Asian seabass (*Lates calcarifer*) nursery farms. Vaccines.

[38] Paankhao N., Sangsawang A., Kantha P., Paankhao S., Promsee K., Soontara C., Kongsriprapan S., Srisapoome P., Kumwan B., Meachasompop P. (2024). Antioxidant and antibacterial efficiency of the ethanolic leaf extract of Kratom (*Mitragyna speciosa* (Korth.) Havil) and its effects on growth, health, and disease resistance against *Edwardsiella tarda* infection in Nile tilapia (*Oreochromis niloticus*). Fish Shellfish Immunol..

[39] Uchiyama M., Mihara M. (1978). Determination of malonaldehyde precursor in tissues by thiobarbituric acid test. Anal. Biochem..

[40] Maehly A. (1954). The assay of catalases and peroxidases. Methods Biochem. Anal..

[41] Jollow D., Mitchell J., Zampaglione N.a., Gillette J. (1974). Bromobenzene-induced liver necrosis. Protective role of glutathione and evidence for 3, 4-bromobenzene oxide as the hepatotoxic metabolite. Pharmacology.

[42] Takada Y., Noguchi T., Okabe T., Kajiyama M. (1982). Superoxide dismutase in various tissues from rabbits bearing the Vx-2 carcinoma in the maxillary sinus. Cancer Res..

[43] Parry R.M., Chandan R.C., Shahani K.M. (1965). A rapid and sensitive assay of muramidase. Proc. Soc. Exp. Biol. Med..

[44] Uchuwittayakul A., Rodkhum C., Srisapoome P. (2025). Production of a monoclonal antibody specific to the IgM heavy chain of Asian seabass (*Lates calcarifer* Bloch, 1790) and its application in assessing health status following vaccination and challenges with *Flavobacterium covae* and *Streptococcus iniae*. Aquaculture.

[45] Vanichavetin K., Uchuwittayakul A., Namdee K., Srisapoome P. (2024). Oral booster effects of bivalent nanovaccine-primed fingerlings of Asian seabass (*Lates calcarifer*, Bloch 1790) to prevent streptococcosis and columnaris diseases. Aquaculture.

[46] Livak K.J., Schmittgen T.D. (2001). Analysis of relative gene expression data using real-time quantitative PCR and the 2^(-Delta Delta C(T)^) method. Methods.

[47] Uchida K., Moriyama S., Breves J.P., Fox B.K., Pierce A.L., Borski R.J., Hirano T., Gordon Grau E. (2009). cDNA cloning and isolation of somatolactin in Mozambique tilapia and effects of seawater acclimation, confinement stress, and fasting on its pituitary expression. Gen. Comp. Endocrinol..

[48] Rattanawongwiboon T., Paankhao N., Buncharoen W., Pansawat N., Kumwan B., Meachasompop P., Kantha P., Pansiri T., Tangthong T., Laksee S. (2025). Characterization and application of synergistically degraded chitosan in aquafeeds to promote immunity, antioxidative status, and disease resistance in Nile tilapia (*Oreochromis niloticus*). Polymers.

[49] Goel M.K., Khanna P., Kishore J. (2010). Understanding survival analysis: Kaplan-Meier estimate. Int. J. Ayurveda. Res..

[50] Wang J., Zhang D., Wang Y., Liu Z., Liu L., Shi C. (2021). Probiotic effects of the *Bacillus velezensis* GY65 strain in the mandarin fish, *Siniperca chuatsi*. Aquac. Rep..

[51] Gao X., Chen A., Zhou Y., Qian Q., Qin L., Tang X., Jiang Q., Zhang X. (2025). Genomic characterization and probiotic potency of *Bacillus velezensis* CPA1-1 reveals its potential for aquaculture applications. Aquaculture.

[52] Yang F., Jiang H., Ma K., Wang X., Liang S., Cai Y., Jing Y., Tian B., Shi X. (2023). Genome sequencing and analysis of *Bacillus velezensis* VJH504 reveal biocontrol mechanism against cucumber *Fusarium* wilt. Front. Microbiol..

[53] Liaqat R., Fatima S., Komal W., Minahal Q., Kanwal Z., Suleman M., Carter C.G. (2024). Effects of *Bacillus subtilis* as a single strain probiotic on growth, disease resistance and immune response of striped catfish (*Pangasius hypophthalmus*). PLoS ONE.

[54] El-Son M.A.M., Elshopakey G.E., Rezk S., Eldessouki E.A.A., Elbahnaswy S. (2022). Dietary mixed *Bacillus* strains promoted the growth indices, enzymatic profile, intestinal immunity, and liver and intestinal histomorphology of Nile tilapia, *Oreochromis niloticus*. Aquac. Rep..

[55] Zhang Y., Yu M., Lin L., Wang J., Zhang D., Wang Q., Elsadek M.M., Wang G., Yao Q., Chen Y. (2022). Effects of dietary *Bacillus velezensis* LSG2-5 on growth, immunity, antioxidant capacity, and disease resistance of Amur minnow (*Rhynchocypris lagowskii* Dybowski). Aquac. Nutr..

[56] Dighiesh H.S., Alharbi N.A., Awlya O.F., Alhassani W.E., Hassoubah S.A., Albaqami N.M., Aljahdali N., Abd El-Aziz Y.M., Eissa E.-S.H., Munir M.B. (2024). Dietary multi-strains *Bacillus* spp. enhanced growth performance, blood metabolites, digestive tissues histology, gene expression of *Oreochromis niloticus*, and resistance to *Aspergillus flavus* infection. Aquac. Int..

[57] Shaheen A.A., Eissa N., Abou-ElGheit E., Yao H., Wang H.-P. (2014). Effect of probiotic on growth performance and growth-regulated genes in yellow perch (*Perca flavescens*). Glob. J. Fish. Aquac. Res..

[58] Vélez-Alavez M., De Anda-Montañez J.A., Galván-Magaña F., Zenteno-Savín T. (2015). Comparative study of enzymatic antioxidants in muscle of elasmobranch and teleost fishes. Comp. Biochem. Physiol. A Mol. Integr. Physiol..

[59] Wang Y., Branicky R., Noë A., Hekimi S. (2018). Superoxide dismutases: Dual roles in controlling ROS damage and regulating ROS signaling. J. Cell Biol..

[60] Georgiou-Siafis S.K., Tsiftsoglou A.S. (2023). The key role of GSH in keeping the redox balance in mammalian cells: Mechanisms and significance of GSH in detoxification via formation of conjugates. Antioxidants.

[61] Zeng Y., Song Z., Song G., Li S., Sun H., Zhang C., Li G. (2025). Oxidative stress and antioxidant biomarker responses in fish exposed to heavy metals: A review. Environ. Monit. Assess..

[62] Ukaegbu K., Allen E., Svoboda K.K.H. (2025). Reactive oxygen species and antioxidants in wound healing: Mechanisms and therapeutic potential. Int. Wound J..

[63] Salimkumar A.V., Sasikumar A.A., Rahman M.S., Elumalai P. (2024). Bacillus Effects on the Immune System. Bacillus Probiotics for Sustainable Aquaculture.

[64] Elbahnaswy S., Elshopakey G.E., Abdelwarith A.A., Younis E.M., Davies S.J., El-Son M.A.M. (2024). Immune protective, stress indicators, antioxidant, histopathological status, and heat shock protein gene expression impacts of dietary *Bacillus* spp. against heat shock in Nile tilapia, *Oreochromis niloticus*. BMC Vet. Res..

[65] Eissa E.-S.H., Abdel Rahman A.N., Ahmed R.A., Hendam B.M., Abd El-Aziz Y.M., Dighiesh H.S., Eissa M.E.H., Okon E.M., Ahmed N.H. (2025). Dietary mixtures of *Bacillus* spp. modulates intestinal morphology, resistance to *Vibrio parahaemolyticus*, response of immune-antioxidant genes, and growth of *Dicentrarchus labrax*. Aquac. Rep..

[66] Mokhtar D.M., Zaccone G., Alesci A., Kuciel M., Hussein M.T., Sayed R.K.A. (2023). Main components of fish immunity: An overview of the fish immune system. Fishes.

[67] Masschalck B., Michiels C.W. (2003). Antimicrobial properties of lysozyme in relation to foodborne vegetative bacteria. Crit. Rev. Microbiol..

[68] Uribe C., Folch H., Enríquez R., Moran G. (2011). Innate and adaptive immunity in teleost fish: A review. Vet. Med..

[69] Zhang Y., Ishikawa M., Koshio S., Yokoyama S., Dossou S., Wang W., Seo S., Chen J., Zheng S., Zhang X. (2024). Effects of dietary supplementation with *Bacillus subtilis natto* on growth, digestive enzyme activity, immune response, and intestinal microorganisms of red sea bream, *Pagrus major*. Fishes.

[70] Giri S., Sukumaran V., Sen S., Jena P. (2014). Effects of dietary supplementation of potential probiotic *Bacillus subtilis* VSG 1 singularly or in combination with *Lactobacillus plantarum* VSG 3 or/and *Pseudomonas aeruginosa* VSG 2 on the growth, immunity and disease resistance of *Labeo rohita*. Aquac. Nutr..

[71] Abdelsamad A.E.M., Said R.E.M., Assas M., Gaafar A.Y., Hamouda A.H., Mahdy A. (2024). Effects of dietary supplementation with *Bacillus velezensis* on the growth performance, body composition, antioxidant, immune-related gene expression, and histology of Pacific white shrimp, *Litopenaeus vannamei*. BMC Vet. Res..

[72] Lee C., Cha J.H., Kim M.G., Shin J., Woo S.H., Kim S.H., Kim J.W., Ji S.C., Lee K.J. (2020). The effects of dietary *Bacillus subtilis* on immune response, hematological parameters, growth performance, and resistance of juvenile olive flounder (*Paralichthys olivaceus*) against *Streptococcus iniae*. J. World Aquac. Soc..

[73] Zou J., Secombes C.J. (2016). The function of fish cytokines. Biology.

[74] Dinarello C.A. (2011). Interleukin-1 in the pathogenesis and treatment of inflammatory diseases. Blood.

[75] Kishimoto T. (2010). IL-6: From its discovery to clinical applications. Int. Immunol..

[76] Matsushima K., Yang D., Oppenheim J.J. (2022). Interleukin-8: An evolving chemokine. Cytokine.

[77] Secombes C.J., Wang T., Austin B. (2012). The innate and adaptive immune system of fish. Infectious Disease in Aquaculture.

[78] Shadrack R.S., Manabu I., Koshio S., Yokoyama S., Zhang Y., Mzengereza K., El Basuini M.F., Dawood M.A. (2022). Effects of single and mixture probiotic supplements on growth, digestive activity, antioxidative status, immune and growth-related genes, and stress response of juvenile red sea bream (*Pagrus major*). Aquac. Nutr..

[79] Jang W.J., Jeon M.H., Lee S.J., Park S.Y., Lee Y.S., Noh D.I., Hur S.W., Lee S., Lee B.J., Lee J.M. (2022). Dietary supplementation of *Bacillus* sp. PM8313 with β-glucan modulates the intestinal microbiota of red sea bream (*Pagrus major*) to increase growth, immunity, and disease resistance. Front. Immunol..

[80] Chang X., Yun L., Liu Z., Shen Y., Feng S., Yang G., Meng X. (2025). Antagonistic effects and the underlying mechanisms of *Bacillus velezensis* and its antibacterial peptide LCI against *Aeromonas hydrophila* Infection in largemouth bass. Probiotics Antimicrob. Proteins.

[81] Yao Y.-Y., Zhou W.-H., Hu J., Yang Y.-L., Li M., Xia R., Ran C., Zhang Z., Zhou Z.-G. (2025). Strain-specific effects of fish originated *Bacillus velezensis* on growth, gut health, and disease resistance of zebrafish. Fish Shellfish Immunol..

[82] Hassanin M.E., El-Murr A., El-Khattib A.R., Abdelwarith A.A., Younis E.M., Metwally M.M.M., Ismail S.H., Davies S.J., Abdel Rahman A.N., Ibrahim R.E. (2024). Nano-*Bacillus amyloliquefaciens* as a dietary intervention in nile tilapia (*Oreochromis niloticus*): Effects on resistance to *Aeromonas hydrophila* challenge, immune-antioxidant responses, digestive/absorptive capacity, and growth. Heliyon.

